# Human Hepatocyte Transplantation: Three Decades of Clinical Experience and Future Perspective

**DOI:** 10.1093/stcltm/szad084

**Published:** 2023-12-16

**Authors:** Jessica Nulty, Hanish Anand, Anil Dhawan

**Affiliations:** Dhawan Lab at the Mowat Labs, Institute of Liver Studies, King’s College London at King’s College Hospital, London, UK; Dhawan Lab at the Mowat Labs, Institute of Liver Studies, King’s College London at King’s College Hospital, London, UK; Dhawan Lab at the Mowat Labs, Institute of Liver Studies, King’s College London at King’s College Hospital, London, UK; Paediatric Liver GI and Nutrition Centre, King’s College Hospital, London, UK

**Keywords:** hepatocyte transplantation, acute liver failure, cell therapy, liver-based metabolic disorders

## Abstract

Orthotopic liver transplantation (OLT) is the current standard of care for both chronic and acute terminal liver disease. However, a major limitation of this treatment is the shortage of healthy donor organs and the need for life-long immunosuppression to prevent graft rejection. Hepatocyte transplantation (HTx) has emerged as a promising, alternative therapeutic approach to either replace OLT or to act as a bridge until a donor liver becomes available thus reducing waiting list mortality. HTx involves the infusion and engraftment of human hepatocytes, typically isolated from organs unsuitable for OLT, into recipient liver parenchyma to carry out the missing hepatic function of the native cells. HTx is less invasive than OLT and can be performed repeatedly if required. The safety of clinical HTx has been shown and treatment results are promising, especially in patients with liver-based metabolic disorders. Nevertheless, HTx has failed to become the standard of care treatment for such disorders. This review aims to evaluate the progress that has been made within the field of HTx over the last 30 years and identify potential shortcomings within the approach which may be hindering its routine clinical application.

## Graphical Abstract

**Graphical Abstract:**
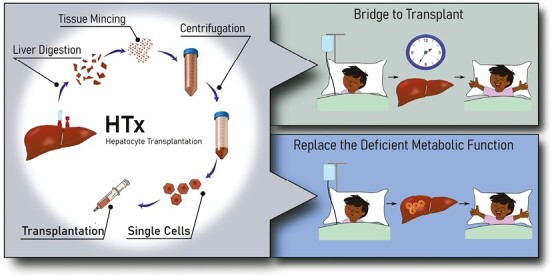

**Significance Statement**

Hepatocyte transplantation (HTx) has shown great promise but has failed to progress toward becoming a routine treatment for liver disease. It involves the infusion and engraftment of human hepatocytes, typically isolated from organs unsuitable for orthotopic liver transplantation (OLT), into recipient liver parenchyma to carry out the missing hepatic function of the native cells. HTx is less invasive than OLT and can be performed repeatedly if required. The safety of HTx has been shown and treatment results are promising, especially in patients with liver-based metabolic disorders. This review includes an overview of HTx and how it has been applied clinically over the last 30 years.

## Introduction

Orthotopic liver transplantation (OLT) is the current standard of care for advanced chronic liver disorders, acute liver failure (ALF), and liver-based single-gene defects. Since the first liver transplantation was performed in 1963, the technique of OLT has been further refined to a standardised procedure involving the resection of the patient’s diseased native liver along with the patient’s retrohepatic inferior vena cava and implanting a healthy liver graft with the interposed donor inferior vena cava followed by 4 vascular anastomoses, hemostasis, and bile duct reconstruction.^1^ However, a major limitation of this treatment is the shortage of healthy donor organs. Furthermore, OLT is a major surgical procedure requiring life-long immunosuppression to prevent graft rejection which in turn increases the risk of infection and other health complications. Hepatocyte transplantation (HTx) has emerged as a promising, alternative therapeutic approach to either replace OLT or to act as a bridge until a donor liver becomes available thus reducing waiting list mortality. HTx involves the infusion and engraftment of human hepatocytes, typically isolated from organs unsuitable for OLT, into recipient liver parenchyma, to carry out the missing hepatic function of the native cells. HTx is less invasive than OLT and can be performed repeatedly if required. Another advantage of HTx is that hepatocytes can be cryopreserved and act as an “off-the-shelf” cellular therapy which can greatly enhance their clinical applicability. In addition, one liver can yield sufficient cell quantities to treat multiple patients from one donor tissue.^2^ The safety of clinical HTx has been shown and treatment results are promising, especially in patients with liver-based metabolic disorders.^3^ Nevertheless, HTx has failed to become the standard of care treatment for such disorders. This review aims to evaluate the progress that has been made within the field of HTx over the last 30 years and identify potential shortcomings within the approach which may be hindering its routine clinical application.

## HTx Clinical Use To Date

The first HTx of autologous hepatocytes was carried out in Japan in 1992 for the treatment of liver cirrhosis.^4^ Since then, HTx has been reported to treat over 100 patients with liver-based disorders worldwide. The most common route of administration for HTx is portal vein infusion either through an indwelling catheter into a branch of the inferior mesenteric vein or through one placed transhepatically under radiographic screening,^5^ however, success has also been demonstrated using intrasplenic and intraperitoneal administration.^6,7^ The therapeutic benefit of HTx varies between the type of liver disease being treated, the pathophysiology of the recipient host environment, and the percentage cell mass necessary to alleviate disease symptoms. For example, the percentage cell mass necessary to provide a therapeutic benefit for liver-based disorders caused by a single gene defect is considerably lower than the amount necessary to carry out the full function of a liver. However, the percentage cell mass refers to the percentage of cells which have engrafted and are fully functional. This does not refer to the number of cells necessary to be infused, which can vary greatly between the type of liver disease being treated as well as numerous other factors such as age of donor cells. Crigler-Najjar syndrome, phenylketonuria, and factor VII deficiency are all liver disorders caused by a single gene defect that leads to a reduced or absent function of the encoded gene product. It has been estimated that a cell engraftment corresponding to ~5%-10% of liver mass is sufficient for clinical benefit of these diseases.^8^ In the case of Crigler-Najjar syndrome, a mutation in the gene UGT1A1 leads to the absence (type 1) or reduced functionality (type 2) of uridine diphosphoglucuronyltransferase and thus the inability to efficiently convert toxic unconjugated bilirubin to its nontoxic conjugated form, leading to a build-up of unconjugated bilirubin in the bloodstream known as unconjugated hyperbilirubinemia.^9-11^ Unconjugated hyperbilirubinemia presents as severe jaundice and can lead to bilirubin encephalopathy and death by kernicterus if left untreated.^9^ Although liver transplantation is the only curative therapy for this disease, HTx has a great potential to substitute the diseased hepatocytes and restore function. In 1995, the first sustained effect of HTx for a single gene defect was demonstrated in a 10-year-old girl who was on the liver transplantation waiting list due to Crigler-Najjar syndrome type I. Prior to HTx treatment, the patient’s serum bilirubin level ranged from 25.5 to 26.6 mg per decilitre, 7.5 × 10^9^ hepatocytes were infused through the portal-vein catheter over a period of 15 hours. Partial correction of hyperbilirubinemia was sustained for 11 months post-transplantation.^12^ Since then, HTx has been used to treat other patients with Crigler-Najjar syndrome as well as numerous other genetic liver diseases such as alpha1-antitryspin deficiency,^13^ familial hypercholesterolemia,^14^ factor VII deficiency,^15^ glycogen storage diseases,^16^ infantile Refsum’s disease,^17^ primary oxalosis,^18^ phenylketonuria,^19-21^ and urea cycle defects^2,22,23^ (Table 1).

**Table 1.** Hepatocyte transplantation: clinical studies in patients with inborn errors of metabolism.

Disease	Patients	Number of cells transplanted	Route of delivery	Cell type	Outcome	Ref.
Crigler-Najjar syndrome type 1	1 female child (10 years)	7.5 × 10^7^	Portal Vein	Fresh primary hepatocytes (5-year-old donor)	OLT after 4 years	^3^
	1 female child (8 years)	7.5 × 10^7^ (9 injections over 5 months)	Portal Vein	Both Fresh and cryopreserved primary hepatocytes	OLT after 20 months	^5^
	1 male child (9 years)	7.5 × 10^7^	Portal Vein	Fresh Primary hepatocytes (47-year-old donor)	OLT after 5 months	^1^
	1 male child (18 months); 1 female child (3 years)	4.3 × 10^7^ 2.1 × 10^7^	Portal Vein	Cryopreserved primary hepatocytes	OLT after 8 months	^2^
	1 female child (8 years)	1.4 × 10^7^	Portal Vein	Fresh primary hepatocytes (7-year-old donor)	OLT after 11 months	^4^
	1 female child (9 year); 1 female child (1 year)	6.1 × 10^7^ (18 infusions from 3 different donors) 2.6 × 10^7^ (14 infusions from 1 donor)	Porth-a-cath in jejunal vein; Broviac in portal vein	Both Fresh and cryopreserved primary hepatocytes	OLT after 6 months OLT after 4 months	^6^
Familial hypercholesterolemia	Five patients (7-41 years)	1.0-3.2 × 10^7^	Portal Vein	Fresh primary hepatocytes transduced through retrovirus-mediated gene transfer for LDLR gene	Variable and transient response	^7^
Factor VII deficiency	1 child (3 months); 1 child (3 years)	1.1 × 10^7^ 2.2 × 10^7^	Portal Vein	Both Fresh and cryopreserved primary hepatocytes	OLT after 7 months OLT after 8 months	^8^
Glycogen storage disease type I	1 female adult (47 years)	2 × 10^7^	Portal Vein	Fresh primary hepatocytes	9 months after trans- plantation, patient on normal diet and can fast for 7 h without experiencing hypoglycaemia	^9^
	1 male adult (18 years)	2 × 10^9^ for first infusion; 1 × 10^9^ for second and 3 × 10^9^ for final infusion	Portal Vein	Both Fresh and cryopreserved primary hepatocytes	250 days after HTx patient on a normal diet	^10^
Infantile Refsum’s disease	1 female child (4 years)	1.1 × 10^9^ for first infusion; 1.4 × 10^8^ and 9 × 10^7^ on day 3, 1.84 × 10^8^ and 2.43 × 10^8^ on day 4, and 1.96 × 10^8^ on day 5	Portal Vein	Both Fresh and cryopreserved primary hepatocytes	Continued metabolic improvement 1 year after HTx	^11^
Progressive familial intrahepatic cholestasis type 2	2 children (18 months and 3 years)	0.2 × 10^7^ 0.4 × 10^7^	Portal Vein	Fresh primary hepatocytes	OLT after 5 months OLT after 14 months	^2^
OTC deficiency	1 male child (5 years)	1 × 10^7^	Portal Vein	Fresh primary hepatocytes	Death 42 days later	^12^
	1 male child (10 hours old)	4 × 10^9^ for first infusion; further 3.3 × 10^9^ between days of life 37 and 51; 1.7 × 10^9^ between days 113 and 116	Portal Vein	Both Fresh and cryopreserved primary hepatocytes	OLT at 6 months	^13^
	1 male child (14 months)	2.4 × 10^7^ 10 infusions over 16 weeks	Portal Vein	Cryopreserved primary hepatocytes	OLT after 6 months	^14^
	1 male child (1 day)	1.74 × 10^9^; 7 infusions over the first month of life and 1 infusion at 5 months.	Portal Vein	Both Fresh and cryopreserved primary hepatocytes	APOLT at 7 months	^15^
	1 male child (6 hours); 1 male child (9 days)	9.4 × 10^8^ in 3 infusions; 8.7 × 10^8^ in 2 infusions	Portal Vein	Cryopreserved primary hepatocytes from one donor (9 days old)	Death at 4 months; Listed for OLT 5 months after HTx	^16^
ASL deficiency	1 female child (3 years)	1.7 × 10^9^ in 7 infusions over 1 month period; 2.5 months after first infusion patent received a further 10 × 10^9^ cells over 2 days; 2 months later a further 1 × 10^9^ cells	Portal Vein	Both Fresh and cryopreserved primary hepatocytes	OLT after 18 months	^17^
CPS1 deficiency	1 male child (10 weeks)	1.87 × 10^9^ over 6 infusions	Portal Vein	Both Fresh and cryopreserved primary hepatocytes	Listed for OLT 7 months after HTx	^16^
Citrullinemia	1 female child (3 years)	1.89 × 10^9^ over 4 infusions	Portal Vein	Both Fresh and cryopreserved primary hepatocytes	Protein intake could be increased 10 months after HTx	^16^

Last year marked the 20-year anniversary of treating our first patient using HTx here at King’s College Hospital, London. In our experience over the last 2 decades, the success of using HTx for liver-based metabolic defects has been varied. When used for the treatment of Crigler-Najjar syndrome, HTx decreased bilirubin between 30% and 50 % for varying lengths of time, in some cases for up to 18 months.^5^ We have shown that HTx can be used in neonates with ornithine transcarbamylase (OTC) deficiency to decrease ammonia levels and successfully bridge the patient to subsequent auxiliary partial orthotopic liver transplantation (APOLT).^24^ We have also shown the transient success of HTx for factor VII deficiency. Hepatocyte infusion improved the coagulation defect and markedly decreased the requirement for exogenous recombinant factor VII (rFVIIa) to approximately 20%.^25^ Furthermore, we have shown that HTx resulted in the clearance of abnormal bile acids and improved the development of a patient with molybdenum deficiency for 18 months.^26^ Despite the relative success of our HTx metabolic program, routine use of HTx for metabolic disorders is hindered by poor engraftment and to date has yet to act as a cure for such disorders but rather acts as a bridge until an appropriate liver graft is available. Although there is merit in using HTx for this purpose, new approaches which can improve hepatocyte engraftment and sustained functionality are needed for HTx to reach its potential as a standard treatment for liver-based metabolic diseases.

HTx has also shown some promising clinical success in the treatment of ALF (Table 2). ALF is a rare condition involving a rapid loss of liver function over the course of days or weeks often in a patient with no pre-existing liver disease. Once ALF progresses in severity, no specific medical treatment exists other than OLT, without which death usually ensues.^27^ This makes HTx a very attractive treatment option whilst the patient waits for a donor liver to become available. HTx has been used to treat various cases of ALFs that have occurred following dilantin,^28^ halothane,^29^ and multiple polysubstance misuse^6,28^ showing promising improvements in encephalopathy and ammonia concentrations. Back in the mid-90s, human foetal HTx was reported for the first time to treat ALF in 7 patients, 5 females, and 2 males with a mean age of ~25 ± 9.5 years. 6 × 10^7^ cells/kg body weight at a conc. of 3 × 10^6^ cells/mL were administered intraperitoneally. The overall survival of the treated group was 43% compared to 33% in matched controls. Furthermore, for the patients who were admitted as encephalopathy grade III, the survival rate was 100% compared to 50% in the control.^7^ Further cases have reported, HTx into the spleen successfully bridged 2 adults for 2 and 10 days until an OLT could be carried out^28^ and a 37-year-old patient with ALF was treated with an intraportal infusion of 8.8 × 10^8^, 96% viable human hepatocytes with immunosuppression fully recovered with a rapid fall in serum ammonia levels.^30^ Biopsies 3 months after showed no detectable trace of donor hepatocytes and immunosuppression was successfully tapered off.

**Table 2.** Hepatocyte transplantation: clinical studies in patients with acute liver failure.

Disease	Patients	Number of cells transplanted	Route of delivery	Cell Type	Outcome	Ref.
Drug-induced liver failure	16 years; 12 years; 10 years	4 × 10^7^−4 × 10^9^	Portal Vein	Cryopreserved primary hepatocytes	Death on day 2; Death on day 7; Death on day 7	^18^
	1 female adult (32 years); 1 male adult (35 years); 1 male adult (55 years)	1.3 × 10^9^; 1 × 10^10^; 3.9 × 10^10^	Intrasplenic	Cryopreserved primary hepatocytes	Death on day 14; Death on day 20; Death in 6 h	^19^
	1 female teenager (13 years); 1 female adult (43 years)	NA	Portal Vein	NA	Death on day 4; Death on day 35	^20^
	1 female adult (27 years); 26 years; 21 years; 35 years; 35 years; 51 years	2.8 × 10^7^; 3 1.2 × 10^9^; 3 infusions of 9 × 10^8^ , 9 × 10^8^ and 2.5 × 10^7^; 5.4 × 10^9^; 3.7 × 10^9^; 3.9 × 10^9^	Intrasplenic; Intrasplenic; Intrasplenic; Portal Vein; Portal Vein; Portal Vein	NA	OLT on day 10; OLT on day 2; Death on day 1; Death on day 18; Full recovery; Death on day 3	^21^
	1 female adult (32 years); 1 male adult (29 years); 1 female adult (20 years); 1 female adult (20 years); 1 female adult (24 years)	60 × 10^6^/kg body weight	Intraperitoneal	Fresh foetal hepatocytes	Death in 30 h; Death in 37 h; Death in 48 h; Full recovery; Full recovery	^22^
Viral-induced acute liver failure	4 years; 54 years	2 infusions of 1.7 × 10^9^; 6.6 × 10^9^	Portal Vein	NA	Death on day 2; Death on day 7	^21^
	1 female adult (29 years); 1 female adult (65 years)	1 × 10^10^; 3 × 10^10^	Portal Vein and Intrasplenic	Cryopreserved primary hepatocytes	Death in 18 h. Death on day 52	^19^
	1 female adult (28 years); 1 female adult (37 years); 1 male adult (43 years)	NA	Intrasplenic; Intrasplenic; Portal Vein	NA	OLT on day 3; Death on day 5; OLT on day 1	^20^
	1 female adult (37 years)	1.2 × 10^8^	Intrasplenic	NA	Full recovery	^23^
	1 female adult (40 years)	60 × 10^6^/kg body weight	Intraperitoneal	Fresh foetal hepatocytes	Death in 13 h	^2^
	1 female adult (40 years)	7.5 × 10^6^	Intrasplenic	cryopreserved primary hepatocytes	Death on Day 4 due to ICP monitor complications	^24^
Idiopathic acute liver failure	3 years; 5 years	4 × 10^9^	Portal Vein	Cryopreserved primary hepatocytes	Full recovery; OLT on day 4	^18^
	3.5 months; 23 years; 48 years	1.8 × 10^8^; 2.86 × 10^8^; 7.5 × 10^8^	Portal Vein; Intrasplenic; Portal Vein	NA	OLT on day 1; OLT on day 5 and death on day 13; Death on day 1	^21^
	1 male child (8 years)	60 × 10^6^/kg body weight	Intraperitoneal	Fresh foetal hepatocytes	Full recovery	^22^
Mushroom-poisoning-induced acute liver failure	1 female (64 years)	8 × 10^9^	Portal Vein	Cryopreserved primary hepatocytes	Full recovery	^25^
Postsurgical acute liver failure	1 male (69 years)	NA	Intrasplenic	NA	Death on day 2	^20^
Acute liver failure induced by acute fatty liver of pregnancy	1 female (26 years)	3 × 10^8^	Intraperitoneal	Fresh foetal hepatocytes	Full recovery	^26^
Alpha 1 anti-trypsin	1 female adult (52 years)	2.2 × 10^7^	Intrasplenic	cryopreserved primary hepatocytes	OLT on Day 2	^24^
TPN/Sepsis	1 male child (6 months)	5.2 × 10^7^	Intrasplenic	cryopreserved primary hepatocytes	Life support stopped on day 7	^24^

Although the use of HTx for the treatment of ALF is promising, the environment into which hepatocytes are being transplanted is very hostile for engraftment and expansion to take place due to the high levels of cellular necrosis and apoptosis.^31^ To clear this cellular debris, macrophages are recruited and activated, in turn secreting transforming growth factor-β (TGFβ) which amplifies injury-induced senescence in hepatocytes.^32^ The severe acute hepatic necrosis present during ALF induces the spread of senescence to remaining viable hepatocytes, thus impairing hepatocyte-mediated regeneration.^32^ This can also affect the transplanted hepatocytes, which may have already experienced stresses during the HTx procedure and are thus more susceptible to cellular senescence and subsequent impairment to their therapeutic effect. For this reason, new strategies are being investigated to evade this harsh microenvironment and prevent hepatocyte senescence such as implanting cells in alternative sites such as the peritoneum^33^ and lymph nodes.^34^ Another approach to protecting transplanted hepatocytes from the harsh microenvironment of a failing liver is to encapsulate the implanted cells into alginate microbeads.^35^ These can be implanted into the peritoneum thus permitting the donor hepatocytes to carry out hepatic functions whilst being protected from the host immune cells. Furthermore, this approach circumvents the need for immunosuppression. The safety of this approach has been demonstrated in 8 pediatric patients with ALF. Of the 8 patients who received an intraperitoneal infusion of microbeads, 4 fully recovered without the need for further treatment, 3 were successfully bridged to OLT, and one patient died.^36^ Given the poor prognosis for children with ALF, these results are very promising for the use of HTx using microbeads. Furthermore, a recent study has shown these types of alginate microbeads containing hepatocytes can be cryopreserved with some maintenance of hepatic functions once thawed, indicating the possibility of an off-the-shelf product being available for rapid treatment of ALF.^37^ In our experience using HTx at King’s College Hospital, some of our greatest successes were a result of using HTX for the treatment of ALF. Currently, there is no proven liver support device available that can bridge the patient to native liver recovery or to transplant so in some cases, HTx is the only option for our patients. Previous clinical experiences transplanting human hepatocytes in ALF have shown limited success when cells were injected either in the liver or the peritoneal cavity, mainly due to rejection and complications associated with the use of immunosuppression in extremely sick patients, which increases the risk of infections. We have developed a technique using liver cells encapsulated in a biocompatible hydrogel to form hepatocyte microbeads.^35^ These microbeads can be infused into the peritoneal cavity of the patient, to temporarily replace the failing liver until regeneration can occur. We have previously treated 8 children and infants with these microbeads.^36^ The technique proved to be safe and, importantly, demonstrated promising efficacy. At the time of treatment, all 8 children met the eligibility for organ transplantation and were awaiting transplant. Four children fully recovered with the treatment thus entirely avoiding the need for liver transplantation and are still doing well, nearly 10 years after the procedure. Given the poor prognosis for children with ALF, these results are very promising for the use of HTx using microbeads. Since then, we have refined our hepatocyte microbead prototype (unpublished). Progressing to a microbead which now involves multiple cell types and an improved hydrogel that better supports the cell function. The new microbeads have shown superior function and longevity in vitro as well as in preclinical studies and are currently being tested in phase I/II clinical trial to show safety and efficacy. Furthermore, a recent study has shown that alginate microbeads can be cryopreserved and still maintain hepatic functions once thawed.^37^ This may enable the production of an off-the-shelf, frozen product which is readily available for the rapid treatment of ALF.

## Increasing Hepatocyte Source

The supply of hepatocytes is limited to the availability of donor organs. As OLT is the current gold standard for most liver-based diseases, any available donor liver will first be assessed for its suitability for transplantation. Only if the tissue is deemed unsuitable for this purpose will it be considered for HTx. As donor availability is already limited, new avenues are being explored to increase the quantity of tissue available for use for HTx such as improving the quality of marginal tissues and accessing underutilised hepatic sources.

### DCD Donors

Livers obtained from donors after cardiac arrest, known as donation after circulatory death (DCD) livers, are often regarded as marginal grafts and are not often used for OLT due to a reduced graft survival rate, higher rate of primary nonfunction, ischemic cholangiopathy, and hepatic artery thrombosis.^38^ Due to excess of demand over the supply of suitable liver tissue, there is a mounting interest in using these DCD livers as a potential source for HTx. Promising results have been shown using various ex vivo graft reconditioning methods including hypothermic^39^ and normothermic^40^liver perfusion systems with oxygenation, extracorporeal membrane oxygenation,^41^ and venous systemic oxygen persufflation using nitric oxide gas.^42^ Here at King’s College Hospital, over the past 10 years, 68% of the livers we have processed for our clinical isolations have been from DCD livers. In our hands, there are no differences between the quality of the cells isolated from DCD compared to livers donated after brainstem death (DBD). Isolated hepatocytes from DCD livers tend to have slightly higher viabilities but this may be due to the circumstances under which we obtain these liver tissues. On average, our DCD livers are from younger donors and also undergo lower cold ischemia times compared to DBD.

### Neonatal Livers

A benefit of HTx is that isolated hepatocytes are not constrained to the same criteria necessary for OLT. In addition to the quality and functionality of the liver parenchymal cells within the graft, the size and architecture of donor tissue are fundamental to successful surgery. For this reason, neonatal donor livers are rarely used for OLT due to the technical difficulties associated with their size such as performing vascular anastomosis. However, neonatal donor livers are an ideal source of hepatocytes for HTx. Studies have shown that hepatocytes isolated from 1- to 23-day-old liver donors showed better post-thawing recovery compared with adult hepatocytes, with better attachment efficiency, cell survival, and a lower number of apoptotic cells.^43^ Furthermore, it has been shown that cell suspensions isolated from neonatal livers contain a higher proportion of hepatic progenitor cells compared to adult hepatocyte suspensions which could contribute to higher levels of regeneration within the liver parenchyma after HTx.^43^ In a recent study, human hepatocytes isolated from neonatal donors after prolonged warm ischemia times were evaluated in an animal model which showed that although neonatal hepatocytes showed signs of immaturity pre-transplantation, implanted hepatocytes undergo maturation in vivo.^44^

We too have seen in our own clinical isolations that hepatocytes isolated from neonatal livers have a higher viability and have higher resistance to the cryopreservation/thawing process and perform well in clinical transplantation.^26^

### Segments From Split Livers

A method often used to expand the available pool of donor organs for OLT is to divide livers into right and left portions that are subsequently implanted into 2 recipients simultaneously. There is much controversy as to where the best line of division is for splitting livers, through Segment IV or through the umbilical fissure. Segment IV receives its blood supply from the left hepatic artery and left portal vein. Often when a donor liver is split between an adult recipient and a pediatric recipient, segment IV is allocated to the right lobe. This can result in a relative ischemia of segment IV which holds the potential risk of sepsis because of infarction.^2^ Due to this risk, here at King’s College Hospital, resection of segment IV used to be standard practice. This excised tissue represents another source of high-quality hepatocytes for HTx. Therefore, in our center, a single donor liver has the potential to benefit 3 separate patients. Similarly, segment I is often not transplanted during traditional OLT using split livers. This may represent another potential source of hepatocytes for HTx. In our experience, however, this type of tissue is better suited for research purposes. The cell yield from this segment is often too low to justify the expense of undergoing a costly cell isolation under good manufacturing practice (GMP) conditions.

### Domino Livers

Another method being explored to increase the supply of donor tissue is domino liver transplantation. Domino liver transplantation utilises explant livers obtained from OLT recipients with single gene mutations (monogenic diseases) as grafts for other patients awaiting transplantation.^45^ These explants are anatomically and functionally normal except for the enzyme defect associated with the monogenic disease, furthermore, non-cirrhotic inherited metabolic liver diseases have shown to be promising source of high-quality hepatocytes for HTx.^46^ The first domino liver was performed in 1995.^47^ An explant was transplanted from a female adult LT recipient with familial amyloidotic polyneuropathy to an advanced oncological patient. Since then, over a thousand domino transplants have been successfully carried out.^45^ Systemic metabolic defects involving multiple organs, such maple syrup urine disease, are the ideal source of domino livers to prevent the possibility of transmitting diseases. This ensures sufficient enzyme activity can be produced in extrahepatic tissues to compensate for the genetic defect being transmitted by the graft. In recent years, the concept of “domino cross-auxiliary liver transplantation” has been developed.^48^ This involves a noncirrhotic inherited metabolic liver diseased liver graft being used for auxiliary partial OLT in another patient with an alternative type of noncirrhotic inherited metabolic liver disease and may significantly increase the donor availability to achieve a mutual compensation for metabolic defects. In terms of HTx, any approach that increases the availability of liver tissues suitable for hepatocyte isolation would thus directly improve the supply of primary hepatocytes. Furthermore, as HTx only replaces a small portion of the liver, any risk of developing symptoms related to metabolic-diseased donor hepatocytes in the recipient is minimal. Therefore, it is feasible to use hepatocytes obtained from explanted livers of patients with various noncirrhotic inherited metabolic liver diseases for domino HTx. The first clinical domino HTx was carried out in 2012.^21^ A 6-year-old boy with severe tetrahydrobiopterin nonresponsive phenylketonuria received hepatocytes which were obtained in part from an explanted glycogen storage type 1b liver. Initial results were promising. Following 2 infusions, blood phenylalanine levels returned within the therapeutic target while the phenylalanine half-life decreased 55%, however, these correcting effects failed to be sustained overtime. Despite the potential of domino HTx, it has not been widely adopted. This may be due to the relatively modest uptake of HTx in general. Current demands are sufficiently met by access to healthy liver tissue. If current shortcomings of HTx are resolved, and its use more widely used, hepatocytes isolated from patients with metabolic liver disease may prove instrumental to meeting demands providing that the metabolic capabilities of the donor and the need of the recipient are accounted for.

## Increasing Hepatocyte Engraftment

Despite the success of HTx as a treatment modality for numerous liver diseases, its long-term efficacy is still hindered by low viability and insufficient engraftment of donor hepatocytes. In an effort to enhance hepatocyte engraftment and reach the 5%-10% of liver cell mass required for clinical benefit, multiple strategies have been carried out to precondition the recipient’s liver and give a selective advantage to the transplanted cells such as partial hepatectomy,^49^ portal embolism,^50^ and liver irradiation.^20^

### Partial Hepatectomy

Liver resection without significant functional impairment may induce a favorable environment for hepatocyte engraftment^51,52^ and has been suggested as a potential pre-treatment to improve the success of HTx.^53^ In the initial cases of HTx carried out for the treatment of familial hypercholesterolemia, autologous tissue was harvested following a left lateral segment sectionectomy. Hepatocytes were subsequently isolated from this tissue and genetically corrected with retroviral transduction of the low-density lipoprotein (LDL) receptor and transplanted back into the liver 3 days post-operation via the portal circulation.^14^ Although this procedure was shown to be safe, engraftment efficiency remained low. Subsequent studies have tried to elucidate the optimal timing of HTx following a hepatectomy. In a more recent study investigating HTx in 2 patients with Crigler-Najjar type 1, a partial hepatectomy was performed to enhance cell engraftment.^49^ This procedure provided a regenerative stimulus as indicated by an increase in hepatocyte growth factor levels. In both patients, the presence of bilirubin glucuronides in bile confirmed graft functionality and serum bilirubin levels decreased by 50%. However, one patient was listed for liver transplant due to a loss of graft function due to a discontinuation of immunosuppression. In the other patient, 6 months post-HTx, serum bilirubin levels progressively increased over a course of 500 days and the patient was listed for liver transplant. This study confirmed the safety of partial hepatectomy and its short-term efficacy in combination with HTX, however could not show an effective long-term benefit of this type of approach.

Although partial hepatectomy may prove beneficial to improve the recipient environment for HTx both endogenous hepatocytes and transplanted hepatocytes can participate in the restoration of liver mass. In animal studies, to further enhance the growth advantage of transplanted hepatocytes, partial hepatectomy has been paired with pyrrolizidine alkaloid retrorsine treatment to disrupt the proliferative capacity of resident hepatocytes.^54,55^

Animal studies have shown that when a host liver is undamaged, transplanted cells can engraft at low levels of efficiency but cannot proliferate. However, when a liver is damaged, whether due to irradiation, inherent metabolic failure, presence of toxic chemicals, or surgical assault, transplanted hepatocytes display a selective growth advantage and are capable of replacing host hepatic tissue.^56,57^

### Irradiation

Another method being investigated to provide a selective proliferative advantage to transplanted hepatocytes is the use of high-dose irradiation of the host liver. Hepatic irradiation in both rodent studies and non-human primate studies has shown that even at low doses (5 Gy), irradiation can cause short-term inhibition of the phagocytic function of Kupffer cells, disrupt the hepatic sinusoidal endothelial barrier, and enhance the overall efficiency of donor hepatocyte engraftment.^58^ Kupffer cells have been associated with the early clearance of transplanted cells following HTx.^59^ By suppressing their phagocytic function by irradiation, transplanted hepatocytes have more time to engraft and may contribute to longer-term survival. The disruption of the hepatic sinusoidal endothelial barrier may contribute to improved hepatocyte engraftment by enabling the entry of donor hepatocytes into the perisinusoidal space of Disse and aiding their migration into the liver plate.^58^ Human trials are currently underway to investigate HTx in combination with hepatic irradiation for Phenylketonuria and acute decompensated liver failure (NCT01345565 and NCT01465100). Initial findings have shown radiation preconditioning to be safe and results in no pathologic changes to the liver, however, 2 of the 3 patients reported experienced early graft loss.^20^ Further retrospective examination indicated that a failure to provide sufficient immunosuppression throughout the initial donor hepatocyte infusion may have initiated graft loss. By using donor-specific CD154+ T-cell immune monitoring to help track the progress of HTx in this study, the third patient included in this irradiated cohort, a 27-year-old with classical phenylketonuria, was closely monitored for graft survival and immunosuppression adjustments could be carried out resulting in a significant decrease of blood phenylalanine levels. Immune monitoring enables the prediction of an increased risk or rejection whilst there is still time to act and may prove to be a useful tool for patient management in future.

### Portal Vein Occlusion

Another method that has been proposed to enhance cell engraftment is partial occlusion of the portal blood flow. Partial occlusion of the portal vein redirects portal blood flow and results in hypertrophy of the nonoccluded liver segments. Portal vein occlusion may be attained through another portal vein ligation or portal vein embolism (PVE). PVE is a technique routinely carried out in clinical surgery prior to large-volume resections in order to induce hypertrophy in the future remnant liver, increasing its size. This has been shown to reduce postoperative morbidity and increase the number of patients amenable to surgery.^60^

Using PVE has been shown to induce hepatocytes proliferation and liver regeneration in rodents,^61,62^ pigs,^63^ and non-human primates.^50,64^ A reversible PVE technique has been described using an absorbable gelatin sponge in a primate model. The resulting temporary embolism lasted for 2 weeks and resulted in an efficient liver regeneration which remained stable for up to a year.^63^ These results were confirmed clinically in the treatment of 20 patients resulting in a hypertrophy ratio of the non-embolized liver of 29% and allowing subsequent surgery in 15 patients.^65^ Furthermore, this technique has shown to be able to be used repeatedly in rodents^66^ and pigs.^67^ This type of technique may be a valuable precondition approach for HTx.

### Emerging Methods for Improving Cell Engraftment

Recent animal studies focused on improving hepatocyte engraftment may provide future promise for human HTx success. One such study used a Hepsin antibody which narrowed the liver sinusoids of recipient mice and thus creating a physical barrier for the transplanted hepatocytes, increasing cell retention.^68^ Hepsin is a type II transmembrane serine protease highly expressed in the liver.^69^ It is involved in the regulation of liver architecture and its depletion leads to enlarged hepatocytes and narrowed liver sinusoids. When heparin antibodies were administered to mice prior to HTx, graft cell numbers increased almost 2-fold in the parenchyma of the recipient livers for up to 20 days after HTx.^68^

The unpredictability and variability of hepatocyte engraftment have prevented the reliable monitoring of donor hepatocyte engraftment and identification of hepatocyte rejection using liver biopsies. Furthermore, the lack of sensitivity in hepatocyte functionality assays such as testing bilirubin and ammonia levels, prevents the diagnosis of donor cell rejection until it is often too late. This has led to the development of novel diagnostic tools to enable earlier detection of rejection which may enable modification to immunosuppressive regimes to prolong HTx therapeutic effects.^20^

Novel methods for tracking transplanted hepatocytes are also needed for cell engraftment to be effectively evaluated. Although numerous successful methods to track cells over time have been established in animal studies, such as transfection/transduction of fluorescent proteins or pre-staining cells with a traceable dye prior to cell transplantation, these methods are not suitable for human use. New methods for tracking cells are emerging across the numerous fields of different cell-based therapies, for example, the use of nanoparticles such as gold nanoparticles and superparamagnetic iron oxide nanoparticles are being investigated to track the fate of transplanted cells in human ophthalmology studies^70^ and for tracking therapeutic cells like chimeric antigen receptor T-cells or tumour-infiltrating lymphocytes in oncology.^71^ In terms of HTx, radioactive indium-111 has been used to label hepatocytes transplanted into a 5-year-old child with OTC deficiency.^72^ The biodistribution of the labelled cells could be analysed non-invasively and resulted in a non-invasive analysis of the biodistribution of transplanted hepatocytes.

### Tolerance

In addition to the successful engraftment of transplanted cells, how well these cells are accepted by the host is also key to the success of the treatment. Antibody-mediated rejection remains a major hurdle for the successful long-term graft survival in all solid organ and cell transplantation. It has been shown that patients with higher levels of donor human leukocyte antigen(HLA)-specific antibodies are at a higher risk of antibody-mediated rejection and graft failure.^73,74^ The liver is often considered an immune-privileged organ. It has a unique blood supply receiving only ~20% of its blood supply from arterial blood, the other 80% of its blood is delivered from the gut via the portal vein.^75^ The latter not only supplies the liver with nutrients from the GI tract but also signalling molecules, microorganisms, toxins, and soluble antigens. Faced with this constant bombardment of possible inflammatory components, the liver has adapted mechanisms to maintain homeostasis and to tightly control inflammatory responses to prevent unnecessary inflammatory reactions.^75^ In terms of transplantation, the liver has long been regarded as an organ with relative resistance to rejection.^76^ Initially it was thought that the tolerogenic properties of hepatocytes would render their immunogenicity much lower than whole organ transplants.^77^ However, it has been shown that allogeneic hepatocytes present as highly antigenic in vivo unlike the liver.^78^ In OLT, the presence of immunologically competent cells, such as antigen presenting cells and other liver cells such as liver sinusoidal endothelial cells (LSECs) and cholangiocytes are thought to play an important role in priming the host immune response and in inducing the development of tolerance.^79^ In HTx, however, these cells are removed during the isolation process and can therefore not induce this immunomodulation. Another explanation that has been proposed by Oldhafer et al is that single-cell hepatocytes may lose their tolerogenic potential when transplanted into a highly inflammatory, allogenic environment.^80^ However, more research is needed to fully understand the mechanisms underlying this phenomenon.

The ideal immunosuppressive protocol for clinical HTx is still unknown, although most centres carry out similar immunosuppressive protocols used for OLT despite the fact that hepatocytes are considerably more immunogenic than the liver itself. These types of immunosuppressive regimes target the immune system in a general manner and thus cause considerable systemic effects such as nephrotoxicity and increased risk for opportunistic infections. An emerging novel strategy for immunosuppression for HTx is to specifically target HLA class I on hepatocytes to reduce alloreactivity. Using lentiviral vectors encoding β2-microglobulin-specific short hairpin RNA the immunogenicity of allografts in HTx was decreased without compromising the metabolic functions of the silenced hepatocytes.^81^

## Need for Advances in Primary Human Hepatocyte (PHH) Cultures and Alternative Cell Sources

**Figure 1.**
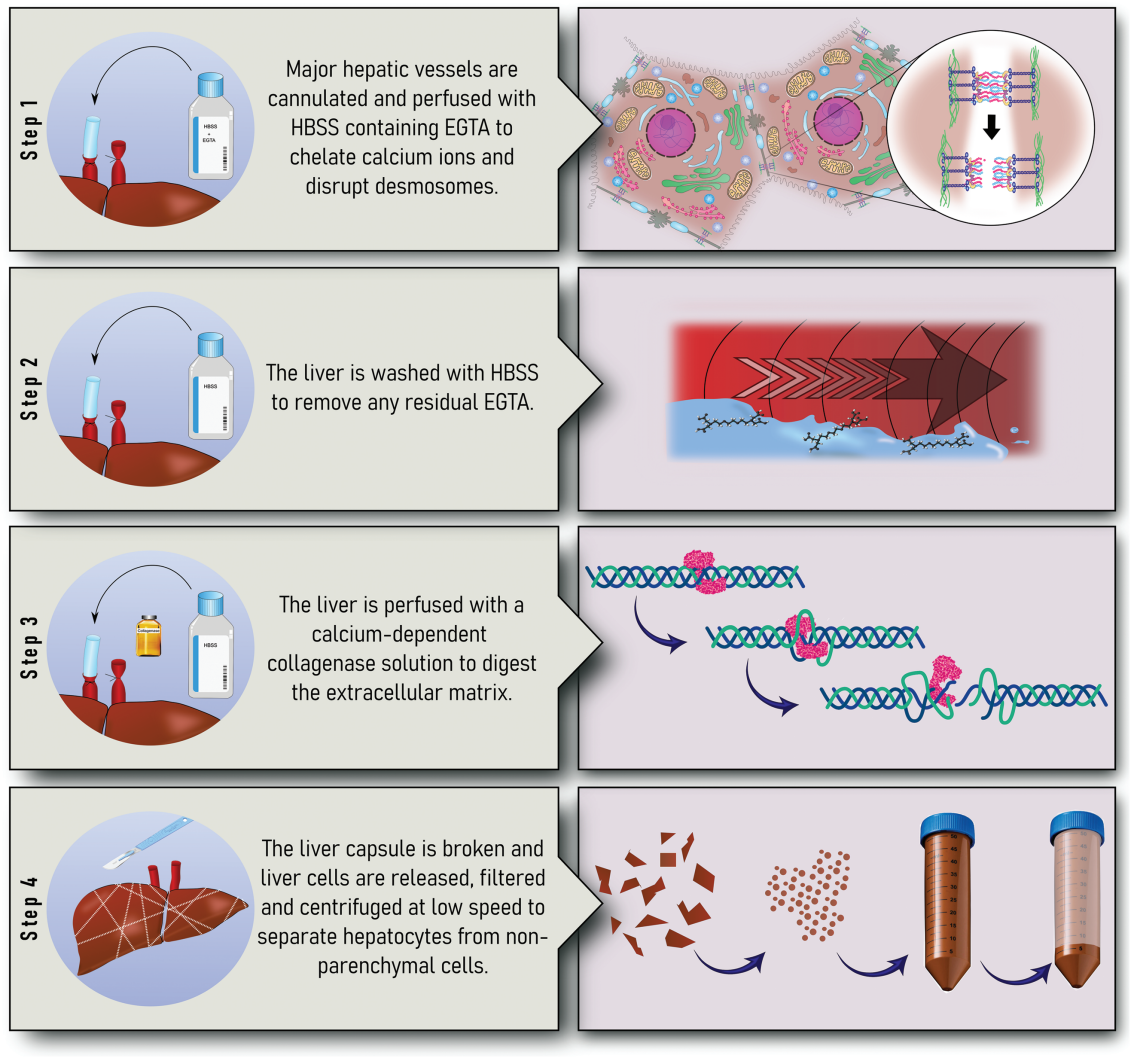
Hepatocyte isolation procedure.

Traditionally hepatocytes used in HTx are isolated from donor livers which have been rejected for OLT due to prolonged warm or cold ischaemia times, mild steatosis, or anatomic disparities. Primary human hepatocytes (PHH) are isolated under GMP in an aseptic and accredited unit using a standardised collagenase perfusion technique that was first developed by Berry and Friend but has since been modified (Fig. 1).^78,79^ The quality of the cells is dependent on the quality of the liver tissue, and as such, can be unpredictable. Isolated PHHs are subsequently used either fresh or cryopreserved at −140 °C until required and are not expanded in vitro compared to other cell types used for other cell-based therapies as they rapidly lose their phenotype in culture and their in vitro expansion has historically been very challenging. Considerable research efforts have bene devoted to overcoming the challenges in PHH cultures and also to find viable alternative cell sources for cell therapies for liver diseases.

Although 2D cultured hepatocytes are suitable for certain short-term assessments such as drug metabolism, within a few days, these hepatocytes dedifferentiate into non-functional cells. 2D cultured cells lack cell-extracellular matrix contact and spatial heterogeneity which are crucial for maintaining long-term functionality in hepatocytes.^80^ 3D methods for culturing can more effectively recapitulate the in vivo environment and facilitate appropriate cell polarity and thus improve cell functionality. In recent years, numerous 3D methods have been used to improve hepatocyte culture. Although traditionally 3D culture systems involve a scaffold to help achieve and maintain a 3D architecture, primary cells maintain the capacity to reform a tissue without such a scaffold. Liver microtissues have been formed through various scaffold-free methods such as using gravity-enforced cellular self-assembly through hanging drops^81^ and low attachment plates^82^ and also through microfluidic systems containing micro-structured surfaces containing arrays of wells which promote assembly of hepatocytes into spheroids.^83^ These types of liver microtissues have been shown to maintain functionality for up to 5 weeks in culture. Scaffold-based 3D culture systems involve varied scaffold materials, many of which are animal-derived such as collagen,^84^ Matrigel,^85^ and decellularised liver tissue.^86^ Often animal-derived products are not fully defined and can have issues with consistent reproducibility issues. This has led to more research into using synthetic polymers for the long-term maintenance of PHH such as peptide-based scaffolds.^87-89^ To date, despite being costly and experiencing batch-to-batch variation, the most commonly used method for 3D culturing of PHH is the pseudo-3D sandwich method which was developed in the early 1990s and involves hepatocytes to be cultured essentially in 2D conditions sandwiched between 2 layers of collagen.^84^

A promising alternative to single-cell HTx comes from the recent breakthrough that liver organoids can be generated in vitro within 3D matrices. Organoids are small, self-organised 3D tissue constructs that can be derived from numerous cell types including embryonic stem cells, primary cells, and induced pluripotent stem cells (iPSCs). The field of organoids is a rapidly expanding field due to its potential as a powerful tool in basic, translational-, and applied-research. Recent advances in the field have led to the generation of human liver organoids from both healthy and diseased tissues, leading to new developments in many aspects of liver development, biology, and disease.^90^ Furthermore, liver organoids generated in vitro exhibit a stable phenotype for over 1 year in culture and have also been shown to engraft in vivo, maintaining functionality, and providing critical liver support.^91,92^

Another emerging approach to produce human hepatocytes is by using chimeric mice with humanised livers. These mice have been specifically engineered to possess a replacement index of 70%-90% after inoculation with human hepatocytes.^93^ These transplanted human hepatocytes undergo extensive proliferation once engrafted and express human albumin, human cytochrome P450 enzymes, and human transporter proteins.^94^ Subsequently, these human hepatocytes may be harvested from mice using well-established collagenase digestion protocols mentioned previously. A similar approach has been carried out in pigs.^95^ Although primarily developed as the first porcine model of hereditary tyrosinemia type 1 (HT1), the authors see the HT1 pigs as a potential in vivo incubator to grow human hepatocytes. Furthermore, the HT1 pig may also be used to grow patient-specific hepatocytes for cell transplantation, avoiding the need for immunosuppression after transplantation.

Recently, it was demonstrated that the addition of a chemical cocktail containing ROCK, TGFβ, and GSK3 inhibitors to the medium mature rodent hepatocytes can enable their long-term in vitro expansion and can convert these cells into bipotent liver progenitor cells with regenerative capacity.^96^ These chemically induced liver progenitors, termed CLiPs, can differentiate into both mature hepatocytes and biliary epithelial cells and were shown to extensively repopulate chronically injured tissue. A similar study showed that a chemically defined culture medium known as transition and expansion media containing growth factors epidermal growth factor (EGF) and hepatocyte growth factor (HGF), agonist of Wnt signaling (CHIR99021), Yap signaling activators lysophosphatidic acid and sphingosine-1-phosphate 6, inhibitor of TGF-β signalling (A83-01), and an inhibitor of ROCK kinase (Y27632) could enable the conversion of mouse hepatocytes to liver progenitor-like cells capable of expansion and passaging over 30 times without evidence of morphological changes.^97^ This type of approach has since been applied to human mature hepatocytes with the development of human hepatocyte medium and a novel human hepatocyte culture system that enables a 10 000-fold expansion of proliferating human hepatocytes (ProliHHs).^98^ With the addition of Wnt3a and removal of Rspo1, Noggin and forskolin in combination with hypoxic culture conditions, ProliHHs are derived. These ProliHHs are at a “bi-phenotypic status” and express markers of both mature hepatocytes and liver progenitors. They retain the functionality of mature PHHs such as plasma protein secretion, glycogen storage, and lipid accumulation and importantly display the remarkable ability to repopulate around 60% of the liver after in vivo transplantation.

### Mesenchymal Stem/Stromal Cells (MSCs)

Mesenchymal Stem/Stromal Cells (MSCs) are multipotent cells with the ability to home to the site of injury and possess potent regenerative and immunomodulatory properties. MSCs are defined by the Society for Cellular Therapy as cells that are plastic-adherent in standard culture conditions; are positive for the markers CD105, CD73, and CD90 and negative for CD45, CD34, CD14 or CD11b, CD79α or CD19, and HLA-DR surface molecules; and possess tripotentiality (can differentiate into osteoblasts, adipocytes, and chondroblasts in vitro).^99^ MSCs can be found in virtually all postnatal tissues including bone marrow,^100^ adipose tissue,^101^ placenta,^102^ and umbilical cord.^103^ MSCs are easily grown in vitro and are therefore an attractive cell source for many cell therapies. Multiple protocols have been reported for the differentiation of MSCs into hepatocytes both in vitro^104,105^ and in vivo.^106^ It has been shown that transplanting MSCs into the fibrotic livers of rodents can improve their liver biochemical profile which Zhang et al^107^ suggest is due to MSC differentiation into functional hepatocytes. However, this concept is debated. Further studies have shown that, although MSCs transplanted into a carbon tetrachloride-induced mouse model of liver fibrosis improved liver function within 2 weeks, very few MSCs underwent hepatocyte trans-differentiation (<3% total liver mass) suggesting MSC-mediated therapeutic effects are driven by mechanisms other than differentiation.^108^ To date, numerous clinical trials have been carried out to investigate the potentiation of MSC transplantation in the treatment of numerous liver diseases including liver cirrhosis,^109-111^ liver failure,^112^ and acute-on-chronic liver failure.^113,114^ These trials have demonstrated the safety of MSC cell therapies and have confirmed their promising potential for the treatment of liver diseases. However, these studies are limited. Larger-scale studies with longer follow-up times are necessary to confirm MSC efficacy in liver disease and more studies are needed to overcome the remaining challenges such as elucidating the best route and timing of administrating and identifying the optimal cell source and dose.

### Human Embryonic Stem Cells (hESCs) and iPSCs

The field of stem cell research has been making huge strides forward since the first characterisation of human embryonic stem cells (hESCs) back in the late 1990s.^115^ The pluripotent capacity of these cells renders them the ability to differentiate into any cell type. Furthermore, in their stem cell state, these cells are capable of practically unlimited self-renewal. Although this bestows enormous therapeutic promise for this cell type, their use has provoked a complex worldwide debate surrounding their ethicality.

These ethical issues appeared to be solved by the breakthrough discovery that somatic cells can be reprogrammed to an embryonic-like state through the introduction of 4 crucial transcription factors including octamer binding transcription factor 3/4 (Oct3/4), sex-determining region Y-box 2 (SRY-Sox2), Krüppel-like factor 4 (Klf4), and cellular-Myelocytomatosis (c-Myc).^116^ These cells were termed iPSCs and have quickly become one of the most studied cell types across scientific literature. iPSCs share many characteristics with hESCs including morphology, pluripotency, self-renewal, and similar gene expression profiles, furthermore, iPSCs preserve the genetic makeup of the host making iPSCs a very powerful tool for disease modeling and regenerative medicine.

To date, numerous protocols have been developed for hepatocyte-like cell (HLC) differentiation from hESCs and iPSCs using a combination of small molecules and growth factors.^117-121^ The majority of protocols consist of a stepwise differentiation process, where hESCs or iPSCs are first primed to definitive endoderm often by using Activin A, FGF2, and BMP4. Next FGF2 and BMP4 are often used for hepatic progenitor specification and finally, HGF and OSM are used for HLC specification. Although HLCs produced using these protocols display many characteristics of PHH, such as albumin secretion, accumulation of glycogen, and urea synthesis, they are often a mixture of foetal and adult phenotypes. In particular, protocols using iPSCs as a starting cell source for HLC differentiation produce cells that more closely resemble foetal or new-born hepatocytes.^122,123^ For hESCs and iPSCs to represent a convenient cell source for HTx, their generation must be standardised to ensure high throughput and high reproducibility of fully differentiated cells and complete elimination of all undifferentiated cells. The latter is particularly important as the transplantation of undifferentiated stem cells carries a high risk of teratoma formation particularly in patients who are immunocompromised/immunosuppressed.^124,125^

## Conclusions

Although over the last 30 years there has been huge progress made within the field of HTx, OLT still remains the gold standard of care for the treatment of liver-based diseases. Despite promising clinical results, particularly in the treatment of metabolic liver disorders, several significant challenges remain to be overcome. The best cell type and culture conditions need to be determined in order to obtain sufficient quality of therapeutic cells at sufficient quantities to meet the demands for large-scale clinical application to succeed. Effective strategies must be identified to improve the engraftment and functionality of the transplanted cells. And lastly, new approaches are needed to improve the host tolerance to transplanted cells thus extending the therapeutic effect of HTx. Strong collaborations between basic researchers, clinicians, and the pharmaceutical industry are vital to close the gap between bench and bedside and translate the scientific breakthroughs into meaningful improvements to the outcomes of liver diseases.

## Data Availability

No new data were generated or analysed in support of this research.

## References

[1] Makowka L , Stieber AC, Sher L, et al. Surgical technique of orthotopic liver transplantation. Gastroenterol Clin North Am. 1988;17(1):33-51. 3292430 PMC3228369

[2] Mitry RR , Dhawan A, Hughes RD, et al. One liver, three recipients: segment IV from split-liver procedures as a source of hepatocytes for cell transplantation. Transplantation. 2004;77(10):1614-1616. 10.1097/01.tp.0000122224.98318.19 15239631

[3] Dhawan A , Puppi J, Hughes RD, Mitry RR. Human hepatocyte transplantation: current experience and future challenges. Nat Rev Gastroenterol Hepatol. 2010;7(5):288-298. Preprint at 10.1038/nrgastro.2010.44 20368738

[4] Mito M , Kusano M, Kawaura Y. Hepatocyte transplantation in man. Transplant Proc. 1992;24(6):3052-3053. 1466053

[5] Dhawan A , Mitry RR, Hughes RD. Hepatocyte transplantation for liver-based metabolic disorders. J Inherit Metab Dis. 2006;29(2-3):431-435. 10.1007/s10545-006-0245-8 16763914

[6] Bilir BM , Guinette D, Karrer F, et al. Hepatocyte transplantation in acute liver failure. Liver Transpl. 2000;6(1):32-40. 10.1002/lt.500060113 10648575

[7] Habibullah CM , Syed IH, Qamar A, Taher-Uz Z. Human fetal hepatocyte transplantation in patients with fulminant hepatic failure. Transplantation. 1994;58(8):951-952. 10.1097/00007890-199410270-00016 7940741

[8] Barahman M , Asp P, Roy-Chowdhury N, et al. Hepatocyte transplantation: quo vadis? Int J Radiat Oncol Biol Phys. 2019;103(4):922-934. 10.1016/j.ijrobp.2018.11.016 30503786 PMC6425726

[9] Crigler JF , Najjar VA. Congenital familial nonhemolytic jaundice with kernicterus. Pediatrics. 1952;10(2):169-180. 12983120

[10] Bosma PJ , Goldhoorn B, Oude Elferink RP, et al. A mutation in bilirubin uridine 5ʹ-diphosphate-glucuronosyltransferase isoform 1 causing Crigler-Najjar syndrome type II. Gastroenterology. 1993;105(1):216-220. 10.1016/0016-5085(93)90029-c 8514037

[11] Tcaciuc E , Podurean M, Tcaciuc A. Management of Crigler-Najjar syndrome. Med Pharm Rep. 2021;94(Suppl No 1):S64-S67. 10.15386/mpr-2234 34527915 PMC8411811

[12] Fox IJ , Chowdhury JR, Kaufman SS, et al. Treatment of the Crigler-Najjar syndrome type I with hepatocyte transplantation. N Engl J Med. 1998;338(20):1422-1426. 10.1056/NEJM199805143382004 9580649

[13] Strom SC , Roy Chowdhury J, Fox IJ. Hepatocyte transplantation for the treatment of human disease. Semin Liver Dis. 1999;19(1):39-48. 10349682 10.1055/s-2007-1007096

[14] Grossman M , Rader DJ, Muller DW, et al. A pilot study of ex vivo gene therapy for homozygous familial hypercholesterolaemia. Nat Med. 1995;1(11):1148-1154. 10.1038/nm1195-1148 7584986

[15] Dhawan A , Mitry RR, Hughes RD, et al. Hepatocyte transplantation for inherited factor VII deficiency. Transplantation. 2004;78(12):1812-1814. 15614156 10.1097/01.tp.0000146386.77076.47

[16] Muraca M , Gerunda G, Neri D, et al. Hepatocyte transplantation as a treatment for glycogen storage disease type 1a. Lancet. 2002;359(9303):317-318. 10.1016/S0140-6736(02)07529-3 11830200

[17] Sokal EM , Smets F, Bourgois A, et al. Hepatocyte transplantation in a 4-year-old girl with peroxisomal biogenesis disease: technique, safety, and metabolic follow-up. Transplantation. 2003;76(4):735-738. 10.1097/01.TP.0000077420.81365.53 12973120

[18] Beck BB , Habbig S, Dittrich K, et al. Liver cell transplantation in severe infantile oxalosis—a potential bridging procedure to orthotopic liver transplantation? Nephrol Dial Transplant. 2012;27(7):2984-2989. 10.1093/ndt/gfr776 22287658

[19] Smets FN , Stephenne X, Debray G, et al. Hepatocyte transplantation transforms severe phenylketonuria to mild hyperphenylalaninemia. Gastroenteroy. 2011;140(5):S-967.

[20] Soltys KA , Setoyama K, Tafaleng EN, et al. Host conditioning and rejection monitoring in hepatocyte transplantation in humans. J Hepatol. 2017;66(5):987-1000. 10.1016/j.jhep.2016.12.017 28027971 PMC5395353

[21] Stéphenne X , Debray FG, Smets F, et al. Hepatocyte transplantation using the domino concept in a child with tetrabiopterin nonresponsive phenylketonuria. Cell Transplant. 2012;21(12):2765-2770. 10.3727/096368912X653255 22889463

[22] Meyburg J , Das AM, Hoerster F, et al. One liver for four children: first clinical series of liver cell transplantation for severe neonatal urea cycle defects. Transplantation. 2009;87(5):636-641. 10.1097/TP.0b013e318199936a 19295306

[23] Stéphenne X , Najimi M, Sibille C, et al. Sustained engraftment and tissue enzyme activity after liver cell transplantation for argininosuccinate lyase deficiency. Gastroenterology. 2006;130(4):1317-1323. 10.1053/j.gastro.2006.01.008 16618422

[24] Puppi J , Tan N, Mitry RR, et al. Hepatocyte transplantation followed by auxiliary liver transplantation—a novel treatment for ornithine transcarbamylase deficiency. Am J Transplant. 2008;8(2):452-457. 10.1111/j.1600-6143.2007.02058.x 18211511

[25] Dhawan A , Mitry RR, Hughes RD, et al. Hepatocyte transplantation for inherited factor VII deficiency. Transplantation. 2004;78(12):1812-1814. 10.1097/01.tp.0000146386.77076.47 15614156

[26] Lee CA , Dhawan A, Iansante V, et al. Cryopreserved neonatal hepatocytes may be a source for transplantation: evaluation of functionality toward clinical use. Liver Transpl. 2018;24(3):394-406. 10.1002/lt.25015 29356341

[27] O’Grady JG , Alexander GJM, Hayllar KM, Williams R. Early indicators of prognosis in fulminant hepatic failure. Gastroenterology. 1989;97(2):439-445. 2490426 10.1016/0016-5085(89)90081-4

[28] Fisher RA , Strom SC. Human hepatocyte transplantation: biology and therapy. In: Berry MN, Edwards AM, eds. The Hepatocyte Review. Springer; 2000:475-501. 10.1007/978-94-017-3345-8_28

[29] Strom SC , Fisher RA, Thompson MT, et al. Hepatocyte transplantation as a bridge to orthotopic liver transplantation in terminal liver failure. Transplantation. 1997;63(4):559-569. 10.1097/00007890-199702270-00014 9047152

[30] Fisher RA , Bu D, Thompson M, et al. Defining hepatocellular chimerism in a liver failure patient bridged with hepatocyte infusion. Transplantation. 2000;69(2):303-307. 10.1097/00007890-200001270-00018 10670643

[31] Dwyer BJ , Macmillan MT, Brennan PN, Forbes SJ. Cell therapy for advanced liver diseases: repair or rebuild. J Hepatol. 2021;74(1):185-199. 10.1016/j.jhep.2020.09.014 32976865

[32] Bird TG , Müller M, Boulter L, et al. TGFβ inhibition restores a regenerative response in acute liver injury by suppressing paracrine senescence. Sci Transl Med. 2018;10(454):eaan1230. 10.1126/scitranslmed.aan1230 30111642 PMC6420144

[33] Nagaki M , Kano T, Muto Y, et al. Effects of intraperitoneal transplantation of microcarrier-attached hepatocytes on D-galactosamine-induced acute liver failure in rats. Gastroenterol Jpn 1990;25(1):78-87. 10.1007/BF02785333 1689685

[34] Hoppo T , Komori J, Manohar R, Stolz DB, Lagasse E. Rescue of lethal hepatic failure by hepatized lymph nodes in mice. Gastroenterology. 2011;140(2):656-666.e2. 10.1053/j.gastro.2010.11.006 21070777 PMC3031768

[35] Mitry RR , Jitraruch S, Iansante V, Dhawan A. Alginate encapsulation of human hepatocytes and assessment of microbeads. Methods Mol Biol. 2017;1506:273-281. 10.1007/978-1-4939-6506-9_19 27830560

[36] Dhawan A , Chaijitraruch N, Fitzpatrick E, et al. Alginate microencapsulated human hepatocytes for the treatment of acute liver failure in children. J Hepatol. 2020;72(5):877-884. 10.1016/j.jhep.2019.12.002 31843649

[37] Jitraruch S , Dhawan A, Hughes RD, et al. Cryopreservation of hepatocyte microbeads for clinical transplantation. Cell Transplant. 2017;26(8):1341-1354. 10.1177/0963689717720050 28901189 PMC5680969

[38] Fondevila C , Hessheimer AJ, Ruiz A, et al. Liver transplant using donors after unexpected cardiac death: novel preservation protocol and acceptance criteria. Am J Transplant. 2007;7(7):1849-1855. 10.1111/j.1600-6143.2007.01846.x 17564639

[39] Dutkowski P , Furrer K, Tian Y, Graf R, Clavien PA. Novel short-term hypothermic oxygenated perfusion (HOPE) system prevents injury in rat liver graft from non-heart beating donor. Ann Surg. 2006;244(6):968-76; discussion 976. 10.1097/01.sla.0000247056.85590.6b 17122622 PMC1856639

[40] Tolboom H , Pouw RE, Izamis ML, et al. Recovery of warm ischemic rat liver grafts by normothermic extracorporeal perfusion. Transplantation. 2009;87(2):170-177. 10.1097/TP.0b013e318192df6b 19155970 PMC2743395

[41] Noormohamed MS , Kanwar A, Ray C, et al. Extracorporeal membrane oxygenation for resuscitation of deceased cardiac donor livers for hepatocyte isolation. J Surg Res. 2013;183(2):e39-e48. 10.1016/j.jss.2013.03.026 23647801

[42] Kageyama S , Yagi S, Tanaka H, et al. Graft reconditioning with nitric oxide gas in rat liver transplantation from cardiac death donors. Transplantation. 2014;97(6):618-625. 10.1097/TP.0000000000000025 24521773

[43] Tolosa L , Pareja-Ibars E, Donato MT, et al. Neonatal livers: a source for the isolation of good-performing hepatocytes for cell transplantation. Cell Transplant. 2014;23(10):1229-1242. 10.3727/096368913X669743 23803290

[44] Bluhme E , Henckel E, Gramignoli R, et al. Procurement and evaluation of hepatocytes for transplantation from neonatal donors after circulatory death. 2022 Jan-Dec;31:9636897211069900. 10.1177/09636897211069900 PMC8811420 35094608

[45] Zhou GP , Sun LY, Zhu ZJ. The concept of “domino” in liver and hepatocyte transplantation. Therap Adv Gastroenterol. 2020;24;13:1756284820968755. 10.1177/1756284820968755 PMC7586492 33149765

[46] Baccarani U , Donini A, Risaliti A, et al. Steatotic versus cirrhotic livers as a source for human hepatocyte isolation. Transplant Proc. 2001;33(1-2):664-665. 10.1016/s0041-1345(00)02191-6 11267006

[47] Furtado A , Tomé L, Oliveira FJ, et al. Sequential liver transplantation. Transplant Proc. 1997;29(1-2):467-468. 10.1016/s0041-1345(96)00206-0 9123085

[48] Qu W , Wei L, Zhu ZJ, et al. Considerations for use of domino cross-auxiliary liver transplantation in metabolic liver diseases: a review of case studies. Transplantation. 2019;103(9):1916-1920. 10.1097/TP.0000000000002602 30801517

[49] Jorns C , Nowak G, Nemeth A, et al. De novo donor-specific HLA antibody formation in two patients with Crigler-Najjar syndrome type I following human hepatocyte transplantation with partial hepatectomy preconditioning. Am J Transplant. 2016;16(3):1021-1030. 10.1111/ajt.13487 26523372 PMC5061095

[50] Dagher I , Boudechiche L, Branger J, et al. Efficient hepatocyte engraftment in a nonhuman primate model after partial portal vein embolization. Transplantation. 2006;82(8):1067-1073. 10.1097/01.tp.0000236103.99456.8f 17060856

[51] Oldhafer F , Wittauer EM, Beetz O, et al. Supportive hepatocyte transplantation after partial hepatectomy enhances liver regeneration in a preclinical pig model. Eur Surg Res. 2021;62(4):238-247. 10.1159/000516690 34044396

[52] Sigal SH , Rajvanshi P, Gorla GR, et al. Partial hepatectomy-induced polyploidy attenuates hepatocyte replication and activates cell aging events. Am J Physiol. 1999;276(5):G1260-G1272. 10330018 10.1152/ajpgi.1999.276.5.G1260

[53] Puppi J , Strom SC, Hughes RD, et al. Improving the techniques for human hepatocyte transplantation: report from a consensus meeting in London. Cell Transplant. 2012;21(1):1-10. 10.3727/096368911X566208 21457616

[54] Laconi E , Oren R, Mukhopadhyay DK, et al. Long-term, near-total liver replacement by transplantation of isolated hepatocytes in rats treated with retrorsine. Am J Pathol. 1998;153(1):319-329. 10.1016/S0002-9440(10)65574-5 9665494 PMC1852941

[55] Tsuchida T , Murata S, Matsuki K, et al. The regenerative effect of portal vein injection of liver organoids by retrorsine/partial hepatectomy in rats. Int J Mol Sci. 2019;21(1):178. 10.3390/ijms21010178 31887985 PMC6981799

[56] Irani AN , Malhi H, Slehria S, et al. Correction of liver disease following transplantation of normal rat hepatocytes into Long–Evans Cinnamon rats modeling Wilson’s disease. Mol Ther. 2001;3(3):302-309. 10.1006/mthe.2001.0271 11273771

[57] Yoshida Y , Tokusashi Y, Lee GH, Ogawa K. Intrahepatic transplantation of normal hepatocytes prevents Wilson’s disease in Long-Evans cinnamon rats. Gastroenterology. 1996;111(6):1654-1660. 10.1016/s0016-5085(96)70029-x 8942746

[58] Yamanouchi K , Zhou H, Roy-Chowdhury N, et al. Hepatic irradiation augments engraftment of donor cells following hepatocyte transplantation. Hepatology. 2009;49(1):258-267. 10.1002/hep.22573 19003915 PMC3416044

[59] Joseph B , Malhi H, Bhargava KK, et al. Kupffer cells participate in early clearance of syngeneic hepatocytes transplanted in the rat liver. Gastroenterology. 2002;123(5):1677-1685. 10.1053/gast.2002.36592 12404242

[60] May BJ , Madoff DC. Portal vein embolization: rationale, technique, and current application. Semin Intervent Radiol. 2012;29(2):81-89. 10.1055/s-0032-1312568 23729977 PMC3444878

[61] Furrer K , Tian Y, Pfammatter T, et al. Selective portal vein embolization and ligation trigger different regenerative responses in the rat liver. Hepatology. 2008;47(5):1615-1623. 10.1002/hep.22164 18395841

[62] Goulinet-Mainot S , Tranchart H, Groyer-Picard MT, et al. Improved hepatocyte engraftment after portal vein occlusion in LDL receptor-deficient WHHL rabbits and lentiviral-mediated phenotypic correction in vitro. Cell Med. 2012;4(2):85-98. 10.3727/215517912X647136 26858856 PMC4733829

[63] Lainas P , Boudechiche L, Osorio A, et al. Liver regeneration and recanalization time course following reversible portal vein embolization. J Hepatol. 2008;49(3):354-362. 10.1016/j.jhep.2008.01.034 18387688

[64] Wilms C , Mueller L, Lenk C, et al. Comparative study of portal vein embolization versus portal vein ligation for induction of hypertrophy of the future liver remnant using a mini-pig model. Ann Surg. 2008;247(5):825-834. 10.1097/SLA.0b013e31816a9d7c 18438120

[65] Tranchart H , Catherine L, Maitre S, et al. Efficient liver regeneration following temporary portal vein embolization with absorbable gelatin sponge powder in humans. J Vasc Interv Radiol. 2015;26(4):507-515. 10.1016/j.jvir.2014.11.033 25640643

[66] Gaillard M , Tranchart H, Lainas P, et al. Improving hepatocyte engraftment following hepatocyte transplantation using repeated reversible portal vein embolization in rats. Liver Transpl. 2019;25(1):98-110. 10.1002/lt.25364 30358068

[67] Gaillard M , Hornez E, Lecuelle B, et al. Liver regeneration and recanalization time course following repeated reversible portal vein embolization in swine. Eur Surg Res. 2020;61(2-3):62-71. 10.1159/000509713 33049754

[68] Hsu YC , Yu IS, Tsai YF, et al. A preconditioning strategy to augment retention and engraftment rate of donor cells during hepatocyte transplantation. Transplantation. 2021;105(4):785-795. 10.1097/TP.0000000000003461 32976366

[69] Li S , Wang L, Sun S, Wu Q. Hepsin: a multifunctional transmembrane serine protease in pathobiology. FEBS J. 2021;288(18):5252-5264. 10.1111/febs.15663 33300264

[70] Mundy DC , Goldberg JL. Nanoparticles as cell tracking agents in human ocular cell transplantation therapy. Curr Ophthalmol Rep. 2021;9(4):133-145. 10.1007/s40135-021-00275-z

[71] Lechermann LM , Lau D, Attili B, Aloj L, Gallagher FA. In vivo cell tracking using PET: opportunities and challenges for clinical translation in oncology. Cancers 2021;13(16):4042. 10.3390/cancers13164042 34439195 PMC8392745

[72] Bohnen NI , Charron M, Reyes J, et al. Use of indium-111-labeled hepatocytes to determine the biodistribution of transplanted hepatocytes through portal vein infusion. Clin Nucl Med. 2000;25(6):447-450. 10.1097/00003072-200006000-00012 10836694

[73] Clerkin KJ , Farr MA, Restaino SW, et al. Donor-specific anti-HLA antibodies with antibody-mediated rejection and long-term outcomes following heart transplantation. J Heart Lung Transplant. 2017;36(5):540-545. 10.1016/j.healun.2016.10.016 27916323 PMC5654313

[74] Kauke T , Oberhauser C, Lin V, et al. De novo donor-specific anti-HLA antibodies after kidney transplantation are associated with impaired graft outcome independently of their C1q-binding ability. Transpl Int. 2017;30(4):360-370. 10.1111/tri.12887 27862352

[75] Allen KJ , Mifsud NA, Williamson R, Bertolino P, Hardikar W. Cell-mediated rejection results in allograft loss after liver cell transplantation. Liver Transpl. 2008;14(5):688-694. 10.1002/lt.21443 18433045

[76] Ibars EP , Cortes M, Tolosa L, et al. Hepatocyte transplantation program: lessons learned and future strategies. World J Gastroenterol. 2016;22(2):874-886. 10.3748/wjg.v22.i2.874 26811633 PMC4716085

[77] Figueiredo C , Oldhafer F, Wittauer EM, et al. Silencing of HLA class I on primary human hepatocytes as a novel strategy for reduction in alloreactivity. J Cell Mol Med. 2019;23(8):5705-5714. 10.1111/jcmm.14484 31180181 PMC6653539

[78] Berry MN , Friend DS. High-yield preparation of isolated rat liver parenchymal cells: a biochemical and fine structural study. J Cell Biol. 1969;43(3):506-520. 4900611 10.1083/jcb.43.3.506 PMC2107801

[79] Mitry RR , Hughes RD, Aw MM, et al. Human hepatocyte isolation and relationship of cell viability to early graft function. Cell Transplant. 2003;12(1):69-74. 10.3727/000000003783985197 12693666

[80] Klaas M , Möll K, Mäemets-Allas K, et al. Long-term maintenance of functional primary human hepatocytes in 3D gelatin matrices produced by solution blow spinning. Sci Rep. 2021;11(1):20165. 10.1038/s41598-021-99659-1 34635750 PMC8505433

[81] Messner S , Agarkova I, Moritz W, Kelm JM. Multi-cell type human liver microtissues for hepatotoxicity testing. Arch Toxicol. 2013;87(1):209-213. 10.1007/s00204-012-0968-2 23143619 PMC3535351

[82] Bell CC , Hendriks DFG, Moro SML, et al. Characterization of primary human hepatocyte spheroids as a model system for drug-induced liver injury, liver function and disease. Sci Rep. 2016;6(1):1-13. 27143246 10.1038/srep25187 PMC4855186

[83] Choi JH , Loarca L, De Hoyos-Vega JM, et al. Microfluidic confinement enhances phenotype and function of hepatocyte spheroids. Am J Physiol Cell Physiol. 2020;319(3):C552-C560. 10.1152/ajpcell.00094.2020 32697600 PMC7509267

[84] Dunn JCY , Tompkins RG, Yarmush ML. Long-term in vitro function of adult hepatocytes in a collagen sandwich configuration. Biotechnol Prog. 1991;7(3):237-245. 1367596 10.1021/bp00009a007

[85] Sidhu JS , Farin FM, Omiecinski CJ. Influence of extracellular matrix overlay on phenobarbital-mediated induction of CYP2B1, 2B2, and 3A1 genes in primary adult rat hepatocyte culture. Arch Biochem Biophys. 1993;301(1):103-113. 10.1006/abbi.1993.1121 8442654

[86] Mazza G , Rombouts K, Rennie Hall A, et al. Decellularized human liver as a natural 3D-scaffold for liver bioengineering and transplantation. Sci Rep. 2015;5(1):1-15. 10.1038/srep13079 PMC4528226 26248878

[87] Macpherson D , Bram Y, Park J, Schwartz RE. Peptide-based scaffolds for the culture and maintenance of primary human hepatocytes. Sci Rep. 2021;11(1):6772. 33762604 10.1038/s41598-021-86016-5 PMC7990934

[88] Wu J , Marí-Buyé N, Muiños TF, et al. Nanometric self-assembling peptide layers maintain adult hepatocyte phenotype in sandwich cultures. J Nanobiotechnol. 2010;12;8:29. 10.1186/1477-3155-8-29 PMC3224541 21143997

[89] Wang S , Nagrath D, Chen PC, Berthiaume F, Yarmush ML. Three-dimensional primary hepatocyte culture in synthetic self-assembling peptide hydrogel. Tissue Eng Part A. 2008;14(2):227-236. 10.1089/tea.2007.0143 18333775

[90] Lee SW , Jung DJ, Jeong GS. Gaining new biological and therapeutic applications into the liver with 3D in vitro liver models. Tissue Eng Regen Med. 2020;17(6):731-745. Preprint at 10.1007/s13770-020-00245-9 32207030 PMC7710770

[91] Takebe T , Sekine K, Enomura M, et al. Vascularized and functional human liver from an iPSC-derived organ bud transplant. Nature. 2013;499(7459):481-484. 10.1038/nature12271 23823721

[92] Rashidi H , Luu NT, Alwahsh SM, et al. 3D human liver tissue from pluripotent stem cells displays stable phenotype in vitro and supports compromised liver function in vivo. Arch Toxicol. 2018;92(10):3117-3129. 10.1007/s00204-018-2280-2 30155720 PMC6132688

[93] Tateno C , Yoshizane Y, Saito N, et al. Near completely humanized liver in mice shows human-type metabolic responses to drugs. Am J Pathol. 2004;165(3):901-912. 10.1016/S0002-9440(10)63352-4 15331414 PMC1618591

[94] Sato Y , Yamada H, Iwasaki K, et al. Human hepatocytes can repopulate mouse liver: histopathology of the liver in human hepatocyte-transplanted chimeric mice and toxicologic responses to acetaminophen. Toxicol Pathol. 2008;36(4):581-591. 10.1177/0192623308318212 18467679

[95] Hickey RD , Mao SA, Glorioso J, et al. Curative ex vivo liver-directed gene therapy in a pig model of hereditary tyrosinemia type 1. Sci Transl Med. 2016;8(349):349ra99. 10.1126/scitranslmed.aaf3838 PMC5477771 27464750

[96] Katsuda T , Kawamata M, Hagiwara K, et al. Conversion of terminally committed hepatocytes to culturable bipotent progenitor cells with regenerative capacity. Cell Stem Cell 2017;20(1):41-55. 10.1016/j.stem.2016.10.007 27840021

[97] Wu H , Zhou X, Fu GB, et al. Reversible transition between hepatocytes and liver progenitors for in vitro hepatocyte expansion. Cell Res. 2017;27(5):709-712. 10.1038/cr.2017.47 28374751 PMC5520858

[98] Zhang K , Zhang L, Liu W, et al. In vitro expansion of primary human hepatocytes with efficient liver repopulation capacity. Cell Stem Cell 2018;23(6):806-819.e4. 10.1016/j.stem.2018.10.018 30416071

[99] Dominici M , Le Blanc K, Mueller I, et al. Minimal criteria for defining multipotent mesenchymal stromal cells The International Society for Cellular Therapy position statement. Cytotherapy. 2006;8(4):315-317. 10.1080/14653240600855905 16923606

[100] Baghaei K , Hashemi SM, Tokhanbigli S, et al. Isolation, differentiation, and characterization of mesenchymal stem cells from human bone marrow. Gastroenterol Hepatol Bed Bench 2017;10(3):208-213. 29118937 PMC5660271

[101] Cao Y , Sun Z, Liao L, et al. Human adipose tissue-derived stem cells differentiate into endothelial cells in vitro and improve postnatal neovascularization in vivo. Biochem Biophys Res Commun. 2005;332(2):370-379. 10.1016/j.bbrc.2005.04.135 15896706

[102] Miao Z , Jin J, Chen L, et al. Isolation of mesenchymal stem cells from human placenta: Comparison with human bone marrow mesenchymal stem cells. Cell Biol Int. 2006;30(9):681-687. 10.1016/j.cellbi.2006.03.009 16870478

[103] Lu LL , Liu YJ, Yang SG, et al. Isolation and characterization of human umbilical cord mesenchymal stem cells with hematopoiesis-supportive function and other potentials. Haematologica. 2006;91(8):1017-1026. 16870554

[104] Ayatollahi M , Soleimani M, Tabei SZ, Kabir Salmani M. Hepatogenic differentiation of mesenchymal stem cells induced by insulin like growth factor-I. World J Stem Cells 2011;3(12):113-121. 10.4252/wjsc.v3.i12.113 22224170 PMC3251745

[105] Snykers S , Vanhaecke T, Papeleu P, et al. Sequential exposure to cytokines reflecting embryogenesis: the key for in vitro differentiation of adult bone marrow stem cells into functional hepatocyte-like cells. Toxicol Sci. 2006;94(2):330-41; discussion 235. 10.1093/toxsci/kfl058 16840566

[106] Iwanaka T , Yamaza T, Sonoda S, et al. A model study for the manufacture and validation of clinical-grade deciduous dental pulp stem cells for chronic liver fibrosis treatment. Stem Cell Res Ther. 2020;11(1):1-19. 32213198 10.1186/s13287-020-01630-w PMC7093986

[107] Zhang GZ , Sun HC, Zheng LB, Guo JB, Zhang XL. In vivo hepatic differentiation potential of human umbilical cord-derived mesenchymal stem cells: therapeutic effect on liver fibrosis/cirrhosis. World J Gastroenterol. 2017;23(46):8152-8168. 10.3748/wjg.v23.i46.8152 29290652 PMC5739922

[108] Chen L , Zhang C, Chen L, et al. Human menstrual blood-derived stem cells ameliorate liver fibrosis in mice by targeting hepatic stellate cells via paracrine mediators. Stem Cells Transl Med. 2017;6(1):272-284. 10.5966/sctm.2015-0265 28170193 PMC5442725

[109] Kharaziha P , Hellström PM, Noorinayer B, et al. Improvement of liver function in liver cirrhosis patients after autologous mesenchymal stem cell injection: a phase I-II clinical trial. Eur J Gastroenterol Hepatol. 2009;21(10):1199-1205. 10.1097/MEG.0b013e32832a1f6c 19455046

[110] Zhang Z , Lin H, Shi M, et al. Human umbilical cord mesenchymal stem cells improve liver function and ascites in decompensated liver cirrhosis patients. J Gastroenterol Hepatol. 2012;27(s2):112-120. 10.1111/j.1440-1746.2011.07024.x 22320928

[111] Suk KT , Yoon JH, Kim MY, et al. Transplantation with autologous bone marrow-derived mesenchymal stem cells for alcoholic cirrhosis: phase 2 trial. Hepatology. 2016;64(6):2185-2197. 10.1002/hep.28693 27339398

[112] Peng L , Xie DY, Lin BL, et al. Autologous bone marrow mesenchymal stem cell transplantation in liver failure patients caused by hepatitis B: short-term and long-term outcomes. Hepatology. 2011;54(3):820-828. 10.1002/hep.24434 21608000

[113] Shi M , Zhang Z, Xu R, et al. Human mesenchymal stem cell transfusion is safe and improves liver function in acute-on-chronic liver failure patients. Stem Cells Transl Med. 2012;1(10):725-731. 10.5966/sctm.2012-0034 23197664 PMC3659658

[114] Lin BL , Chen JF, Qiu WH, et al. Allogeneic bone marrow–derived mesenchymal stromal cells for hepatitis B virus–related acute-on-chronic liver failure: a randomized controlled trial. Hepatology. 2017;66(1):209-219. 10.1002/hep.29189 28370357

[115] Thomson JA , Itskovitz-Eldor J, Shapiro SS, et al. Embryonic stem cell lines derived from human blastocysts. Science. 1998;282(5391):1145-1147. 10.1126/science.282.5391.1145 9804556

[116] Takahashi K , Yamanaka S. Induction of pluripotent stem cells from mouse embryonic and adult fibroblast cultures by defined factors. Cell. 2006;126(4):663-676. 10.1016/j.cell.2006.07.024 16904174

[117] Hay DC , Zhao D, Fletcher J, et al. Efficient differentiation of hepatocytes from human embryonic stem cells exhibiting markers recapitulating liver development in vivo. Stem Cells. 2008;26(4):894-902. 10.1634/stemcells.2007-0718 18238852

[118] Si-Tayeb K , Noto FK, Nagaoka M, et al. Highly efficient generation of human hepatocyte-like cells from induced pluripotent stem cells. Hepatology. 2010;51(1):297-305. 10.1002/hep.23354 19998274 PMC2946078

[119] Basma H , Soto-Gutiérrez A, Yannam GR, et al. Differentiation and transplantation of human embryonic stem cell-derived hepatocytes. Gastroenterology. 2009;136(3):990-999. 10.1053/j.gastro.2008.10.047 19026649 PMC2732349

[120] Hannan NRF , Segeritz CP, Touboul T, Vallier L. Production of hepatocyte-like cells from human pluripotent stem cells. Nat Protoc. 2013;8(2):430-437. 23424751 10.1038/nprot.2012.153 PMC3673228

[121] Zhou Q , Xie X, Zhong Z, Sun P, Zhou X. An efficient method for directed hepatocyte-like cell induction from human embryonic stem cells. J Vis Exp. 2021;2021(171):e62654. 10.3791/62654 34028444

[122] Baxter M , Withey S, Harrison S, et al. Phenotypic and functional analyses show stem cell-derived hepatocyte-like cells better mimic fetal rather than adult hepatocytes. J Hepatol. 2015;62(3):581-589. 10.1016/j.jhep.2014.10.016 25457200 PMC4334496

[123] Raju R , Chau D, Notelaers T, et al. In vitro pluripotent stem cell differentiation to hepatocyte ceases further maturation at an equivalent stage of e15 in mouse embryonic liver development. Stem Cells Dev. 2018;27(13):910-921. 10.1089/scd.2017.0270 29851366 PMC6916117

[124] Hentze H , Soong PL, Wang ST, et al. Teratoma formation by human embryonic stem cells: Evaluation of essential parameters for future safety studies. Stem Cell Res. 2009;2(3):198-210. 10.1016/j.scr.2009.02.002 19393593

[125] Gabriel E , Schievenbusch S, Kolossov E, et al. Differentiation and selection of hepatocyte precursors in suspension spheroid culture of transgenic murine embryonic stem cells. PLoS One. 2012;7(9):e44912. 10.1371/journal.pone.0044912 23028675 PMC3454367

